# Diabetes and brain: omics approaches to study diabetic encephalopathy

**DOI:** 10.3389/fendo.2025.1570585

**Published:** 2025-05-12

**Authors:** Nicoletta Lionetti, Maria Grazia Di Lago, Tania Brescia, Federica Bevilacqua, Antonio Gnoni

**Affiliations:** Department of Basic Medical Sciences, Neuroscience and Sense Organs, Section of Clinical Biochemistry, University of Bari “Aldo Moro”, Bari, Italy

**Keywords:** diabetic encephalopathy, omics approaches, oxidative stress, neuroinflammation, energy metabolism

## Abstract

Diabetes mellitus (DM) is a complex metabolic disorder associated with many complications, including diabetic encephalopathy (DE). DE is a severe neurological condition characterized by a progressive decline in cognitive and motor functions, significantly impacting patients’ quality of life. Despite advancements in understanding DM, the intricate pathogenetic mechanisms underlying DE remain incompletely elucidated. This review comprehensively analyzes the application of omics technologies to decipher the molecular basis of DE and identify potential diagnostic biomarkers and therapeutic targets. Several studies on animal models of DE have revealed specific metabolic signatures and changes in gene expression in key memory brain regions, like the hippocampus, highlighting potential therapeutic targets. We explore how these “omics” approaches have provided novel insights into the complex interplay of factors contributing to DE. Recurrent alterations were identified upon evaluation of analysis from human tissues and *in vitro* models of DE. Findings indicate that this pathological condition is characterized by impaired energy metabolism, oxidative stress, neuroinflammation, neuroendocrine dysfunction and the influence of the gut microbiota. A multi-omics approach, integrating data from various models and limited human studies, enhances translational understanding of DE pathogenesis, with new implications for diagnosis and treatment.

## 1 Introduction

Diabetes mellitus (DM) is a complex metabolic disorder. Among its complications, diabetic encephalopathy (DE) represents a severe and increasingly prevalent neurological condition, characterized by a progressive decline in cognitive and motor functions, significantly impacting patients’ quality of life. DM primarily manifests in two forms: type 1 DM (T1DM), caused by autoimmune destruction of pancreatic beta cells, and type 2 DM (T2DM), characterized by insulin resistance (IR). Both forms involve impaired glycemic control, triggering alterations in lipid and protein metabolism, consequently leading to elevated blood lipid levels (1). Both chronic hyperglycemia and hyperlipidemia exert long-term deleterious effects on various organs and systems, including the kidneys, eyes, neurons, heart, and blood vessels (2–6). The persistence of the diabetic condition can lead to the development of DE, characterized, as mentioned, by a progressive decline in cognitive and motor abilities.

Clinically, DE manifests with a spectrum of symptoms ranging from mild cognitive impairments, such as difficulties with concentration, memory, and decision-making, to more severe manifestations like dementia (7). Motor dysfunctions, including difficulties with gait and coordination, can also be observed, potentially correlated with diabetic neuropathy and vascular complications (8, 9).

Mood alterations (such as irritability and depression) and, in some cases, language difficulties can also be present (10). The diagnosis of DE is often complex, as there is no single definitive test. It relies on a comprehensive clinical evaluation, including medical history, neurological examination, cognitive assessments, and exclusion of other possible causes of cognitive decline through blood tests, neuroimaging (CT scans or MRI), and other relevant investigations (11).

The precise pathological mechanisms underlying DE are not yet fully elucidated but are believed to be multifactorial, involving chronic hyperglycemia induced by diabetes, insulin deficits leading to complex metabolic dysfunctions, neuroinflammation, oxidative stress, and hyperleptinemia (12). These interconnected factors contribute to mitochondrial dysfunctions, apoptosis, neuronal atrophy, altered neurotransmitter release, and synaptic reorganization, culminating in impaired brain performance (13). At the neuronal level, DM is also associated with phenomena such as increased neuronal apoptosis, which further contributes to neuronal loss and accelerated atrophy, causing chronic brain lesions in diabetes, leading to a decrease in cognitive and mental functions, with consequent loss of performance and quality of life (14). Currently, oxidative and nitrosative stress are considered a universal mechanism for the development of all DM complications (15). Although it plays a role in the pathogenesis of T2DM, it is crucial to recognize that DE pathogenesis involves a complex interplay of metabolic, inflammatory, and neuroendocrine factors. Indeed, hyperglycemia can increase intracellular oxidative stress through various mechanisms, including the inhibition of endothelial nitric oxide (NO) synthesis (16, 17).

**Figure 1:**
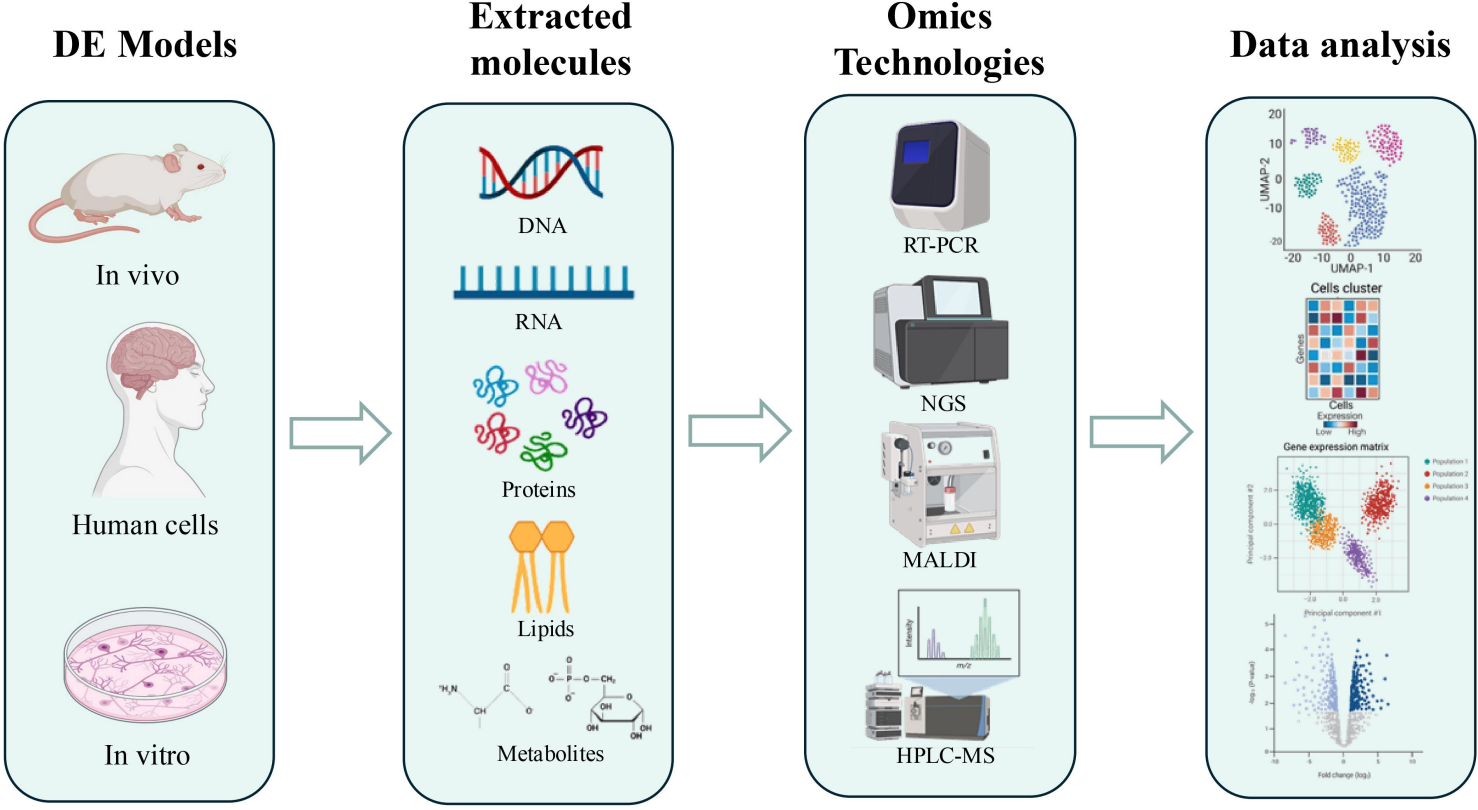
Schematic representation of the workflow of omics approaches for the study of DE. The DE models used are primarily murine, but also include human tissues and cell lines. Extraction of molecules of interest is performed depending on the approach used: nucleic acids for genomics and transcriptomics, proteins for proteomics, lipids for lipidomics, and metabolites for metabolomics. Only some of the techniques used, such as PCR, RNAseq, MALDI, and HPLC-MS, are illustrated, followed by an illustration of data analysis. Figure generated using app.biorender.com.

However, the complexity of the pathogenetic mechanisms involved in DE necessitates a global and integrated study approach, where omics sciences assume a role of primary importance. Omics represent a set of biological disciplines aimed at the comprehensive and large-scale analysis of the molecular components of a biological system. This work considers the application of omics approaches to study nucleic acids, proteins, lipids and metabolites extracted from animal and *in vitro* models of DE, as well as from patient tissue (**Figure 1**). Specifically, genomics studies the entire genome of an organism, identifying genetic variations that may predispose to DE or influence its progression (18). In the study of DE, given the absence of well-defined target genes, research has instead focused on transcript analysis to identify a “molecular signature” of the pathology. Transcriptomics analyzes the complete set of RNA molecules (transcriptome), providing information on gene expression and biological pathways active in the brain under diabetic conditions (19). Advanced technologies such as RNA sequencing (RNA-Seq), single-cell RNA sequencing (scRNA-Seq), and quantitative PCR (qPCR) have uncovered dysregulated pathways associated with inflammation, apoptosis, mitochondrial dysfunction, and blood-brain barrier integrity (20). Furthermore, regulatory molecules such as microRNAs (miRNAs), long non-coding RNAs (lncRNAs), and circular RNAs (circRNAs) are emerging as key modulators of gene expression in DE. Recent studies have highlighted the role of m6A methylation in tau protein hyperphosphorylation, a hallmark of diabetic neurodegeneration, offering novel insights for diagnostic and therapeutic approaches (21). Proteomics studies the entire set of proteins (proteome), analyzing their abundance, modifications, and interactions, to understand how diabetes influences proteins function in the brain (22). Approaches such as mass spectrometry, including MALDI-ToF and MS/MS coupled with liquid chromatography, have identified specific proteins and their functional variants (proteoforms), expanding our understanding of protein complexity in DE. Both bottom-up and top-down techniques have revealed the role of proteoforms in cellular dysfunction, while emerging methods such as GeLC-MS/MS and N,N’-Bis(Acryloyl)Cystamine (BAC)-crosslinked gels are enhancing analytical efficiency, accelerating biomarker and therapeutic target discovery (23, 24). Complementing proteomics, lipidomics analyzes the complete set of lipids (lipidome) in biological systems, providing insights into lipid metabolism, signaling pathways, and membrane integrity, all of which are relevant to DE pathology (25). Metabolomics analyzes the complete set of metabolites (metabolome), providing a snapshot of the metabolic state of the brain and identifying metabolic alterations associated with DE (22). High-resolution metabolite analysis, enabled by techniques such as nuclear magnetic resonance (NMR) and mass spectrometry imaging, has identified alterations in key metabolic pathways involved in DE, including glucose, lipid, and amino acid metabolism. Metabolomics is emerging as a critical tool for prevention, early diagnosis, and personalized therapies, offering new opportunities to address the clinical burden associated with DE (22, 26).

The integration of data obtained from the different omics studies is fundamental to achieve a holistic and complete view of DE pathogenesis (**Table 1**). This “multi-omics” approach allows the identification of complex molecular networks and to understand how alterations at the level of genes, RNA, proteins, and metabolites interact with each other in determining cognitive decline. Given the impossibility of obtaining brain tissue samples from living patients, DE research mainly relies on *in vitro* (cell lines) and *in vivo* (animal models). Cell lines allow the study of specific cellular mechanisms in response to diabetic stimuli, while animal models replicate some pathophysiological aspects of human DE, allowing the study of brain alterations *in vivo* and the testing of potential therapeutic interventions. This review aims to analyze how omics approaches have advanced our understanding of the molecular origins of DE, focusing on the identification of novel biomarkers and potential therapeutic targets, moving beyond a sole focus on oxidative stress to encompass a broader perspective of the molecular mechanisms involved in this complex condition.

**Table 1:** Omics studies on DE models regarding the changes of mRNA, protein, lipids and metabolites.

Article Title	Omics Approach	Technique	Model	Results
Han et al. (27) Hippocampal transcriptome analysis reveals mechanisms of cognitive impairment in beagle dogs with type 1 diabetes	Transcriptomics	RNA-Seq	Diabetic dogs	Identified DEGs related to metabolic disorders and cognitive dysfunction in the hippocampus. ↑ DBH, ↑ IGFBP2, ↑ AVPR1A, ↑ DRAXIN
Also Liu et al. (28) The circRNA circ-Nbea participates in regulating diabetic encephalopathy	Transcriptomics	RNA-Seq	db/db mice	Circ-Nbea has been identified as a potential key regulator in the development of DE, mediated by its interaction with miR-128-3p.
Liu et al. (28)The circRNA circ-Nbea participates in regulating diabetic encephalopathy	Transcriptomics	RNA-Seq	db/db mice	Identified circRNAs, including circ-Nbea, potentially involved in DE pathogenesis. ↓ circo-Nbea
Qin et al. (29) Taohe Chengqi decoction improves diabetic cognitive dysfunction by alleviating neural stem cell senescence through HIF1α-driven metabolic signaling	Transcriptomics	RNA-Seq	HFD/STZ-induced diabetic mice	Analyzed gene expression changes in neural stem cells related to cellular senescence.
Castillo-Armengol et al. (30) Disrupted hypothalamic transcriptomics and proteomics in a mouse model of type 2 diabetes exposed to recurrent hypoglycemia	Transcriptomics	Single-nuclei RNA-seq	Diabetic mice with recurrent hypoglycemia	Analyzed changes in hypothalamic transcriptome, highlighting decreased genes involved in oxidative phosphorylation. ↓ Genes involved in oxidative phosphorylation, ↓ Expression of vacuolar H+- and Na+/K+-ATPases
Ma et al. (31) Single-Cell Sequencing Analysis of the db/db Mouse Hippocampus Reveals Cell-Type-Specific Insights Into the Pathobiology of Diabetes-Associated Cognitive Dysfunction	Transcriptomics	scRNA-seq	db/db mice	Revealed microglial activation and a pro-inflammatory phenotype in DE.
Wang et al. (32) Control of macrophage autophagy by miR-384-5p in the development of diabetic encephalopathy	Transcriptomics	miRNA analysis	STZ-induced diabetic mice	Investigated the miR-384-5p/Beclin-1 axis in macrophage autophagy in DE.
Xiang Q. et al. (33) Heterogeneity and synaptic plasticity analysis of hippocampus based on db-/- mice induced diabetic encephalopathy	Transcriptomics	Single-cell RNA-seq	Diabetic animal model	Identified distinct sub-clusters of OPCs with unique gene expression profiles.
Qu M. et al. (21) High glucose induces tau hyperphosphorylation in hippocampal neurons via inhibition of ALKBH5-mediated Dgkh m^6^A demethylation: a potential mechanism for diabetic cognitive dysfunction.	Transcriptomics	m6A-mRNA epitranscriptomic microarray	STZ-induced diabetic rats	Demonstrated that high glucose inhibits ALKBH5-mediated Dgkh m6A demethylation, leading to tau hyperphosphorylation.
Shi et al. (34) Decreased miR-132 plays a crucial role in diabetic encephalopathy by regulating the GSK-3β/Tau pathway	Transcriptomics	HT-22 cells/Sprague Dawley (SD)	HFD/STZ-induced T2DM rats	MiR-132 exerts protective effects from DE injury by repressing GSK-3β expression and alleviating Tau hyperphosphorylation in HT-22 cells and hippocampus tissues.
Peng W (35). Vof16-miR-205-Gnb3 axis regulates hippocampal neuron functions in cognitively impaired diabetic rats	Transcriptomics	miRNA analysis	STZ-induced diabetic rats	Showed that decreased miR-132 plays a crucial role in DE by regulating the GSK-3β/Tau pathway.
Fu et al. (36) Exploring the pathogenesis of diabetic encephalopathy based on the ceRNA regulatory network of lncRNA-miRNA-mRNA	Transcriptomics	RNA-Seq	GK-/- diabetic rats	Studied the competitive endogenous RNA network involving lncRNA-miRNA-mRNA interactions in DE pathogenesis.
Li X. et al. (37) Proteome analysis of differential protein expression in brain of rats with type 1 diabetes mellitus	Proteomics	2-DE, MALDI-TOF-MS	T1DM rats	Identified 8 differentially expressed proteins, including myosin light chain kinase (MLCK).
Taurino F. et al. (38) Mitochondrial proteome analysis reveals depression of the Ndufs3 subunit and activity of complex I in diabetic rat brain	Proteomics	2D-DIGE, LC-MS/MS	STZ-induced T1DM rats	Discovered a significant reduction in Ndufs3 in mitochondrial Complex I.
Zhang N. et al. (39) Inhibition of YIPF2 Improves the Vulnerability of Oligodendrocytes to Human Islet Amyloid Polypeptide	Proteomics	Western blotting and co-immunoprecipitation	Murine cell culture of oligodendrocytes and neurons	Found that hIAPP significantly reduced the expression of CD147, disrupting lactate transport.
Calábria L.K. et al. (40) Overexpression of myosin-IIB in the brain of a rat model of streptozotocin-induced diabetes	Proteomics	Affinity chromatography, MALDI-TOF-MS	STZ-induced diabetic rats	Isolated and investigated calmodulin-binding proteins (CaMBPs) in the hippocampus.
LU M. et al. (41) Protective effects of grape seed proanthocyanidin extracts on cerebral cortex of streptozotocin-induced diabetic rats through modulating AGEs/RAGE/NF-kappaB pathway	Proteomics	RT-PCR/western blot	Diabetic rats	Highlighted the importance of AGEs/RAGE/NF-kB p65 pathway in DE and the protective effects of GSPE.
Wenwen Yu et al. (42) The attenuation effect of potassium 2-(1-hydroxypentyl)-benzoate in a mouse model of diabetes-associated cognitive decline: The protein expression in the brain	Proteomics	2D-DIGE, LC-LTQ-MS/MS	KK-Ay mice	Analyzed protein expression in the brains of mice treated with dl-PHPB, identifying several altered proteins.
Wei W. et al. (43) Quantitative Proteomics Characterization of the Effect and Mechanism of Trichostatin A on the Hippocampus of Type II Diabetic Mice	Proteomics	DIA - MS	db/db mice	Analyzed hippocampal proteins, revealing 174 differentially expressed proteins between db/db and db/m mice.
Wang ST. et al. (44) Gadolinium Retention and Clearance in the Diabetic Brain after Administrations of Gadodiamide, Gadopentetate Dimeglumine, and Gadoterate Meglumine in a Rat Model	Proteomics	ICP-MS/MS	STZ-induced diabetic rats	Found that diabetic rats clear GBCA from their system more quickly than healthy rats.
Diviccaro et al. (45) Neuroactive Steroid–Gut Microbiota Interaction in T2DM Diabetic Encephalopathy	Proteomics	LC-MS/MS	ZDF rats	Investigated the correlation between neuroactive steroids, gut microbiota, and stress hormones in DE.
Cermenati G. et al. (46) Diabetes alters myelin lipid profile in rat cerebral cortex: Protective effects of dihydroprogesterone	Lipidomics	FIA-MS/MS, GC-MS	STZ-induced diabetic rats	Quantified total fatty acids and cholesterol in myelin, finding significant alterations in myelin lipid composition.
Huo M. et al. (47) Spatially Resolved Metabolomics Based on Air-Flow-Assisted Desorption Electrospray Ionization-Mass Spectrometry Imaging Reveals Region-Specific Metabolic Alterations in Diabetic Encephalopathy	Metabolomics	Mass spectrometry imaging AFADESI-MSI	HFD/STZ-induced diabetic rats	Identification of 19 discriminatory metabolites, including glucose-6-phosphate, succinic acid and taurine, with significant alterations in brain regions such as hippocampus, cortex and thalamus.
Cheng et al. (48) Mass Spectrometry Imaging Reveals Spatial Metabolic Alterations and Salidroside’s Effects in Diabetic Encephalopathy	Metabolomics	Mass spectrometry imaging	db/db mice	Identified significant changes in glucose-6-phosphate, glutamine, adenosine, and L-carnitine in different brain regions.
Meng et al. (49) Mapping of Fatty Aldehydes in the Diabetic Rat Brain Using On-Tissue Chemical Derivatization and Air-Flow-Assisted Desorption Electrospray Ionization-Mass Spectrometry Imaging	Metabolomics	Mass spectrometry imaging	HFD/STZ-induced diabetic rats	Found a significant increase in fatty aldehydes (FALs) in vulnerable brain areas.
Chen et al. (50) A novel hippocampus metabolite signature in diabetes mellitus rat model of diabetic encephalopathy	Metabolomics	GC-MS	STZ-induced diabetic rats	Identified a specific metabolic signature in the hippocampus, characterized by a reduction in N-acetyl aspartate and dihydroxyacetone phosphate.
Romano et al. (51) Short-term effects of diabetes on neurosteroidogenesis in the rat hippocampus	Metabolomics	LC-MS/MS	STZ-induced diabetic rats	Observed a significant reduction in neurosteroids such as pregnenolone and allopregnanolone. Allopregnanolone is more reduced in diabetic female rats
Pesaresi et al. (52) Sex differences in neuroactive steroid levels in the nervous system of diabetic and non-diabetic rats	Metabolomics	LC-MS/MS	STZ-induced diabetic rats	Found significant differences between males and females in the levels of neuroactive steroids.
Zhuang et al. (53) Huang-Lian-Jie-Du Decoction alleviates diabetic encephalopathy by regulating inflammation and pyroptosis via suppression of AGEs/RAGE/NF-κB pathways	Metabolomics	UPLC-Q- Orbitrap HRMS/MS	HFD/STZ- induced T2DM rats	Showed that HLJDD improves cognitive function and regulates phospholipid, fatty acid, and glucose metabolism.
He et al. (54) Explore of the beneficial effects of Huang-Lian-Jie-Du Decoction on diabetic encephalopathy in db/db mice by UPLC-Q-Orbitrap HRMS/MS based untargeted metabolomics analysis.	Metabolomics	Untargeted metabolomics analysis UPLC-Q- Orbitrap HRMS/MS	db/db mice	Showed that HLJDD restores the levels of 11 key metabolites involved in processing fats, glucose, and antioxidants.
Gao et al. (55) Type 1 diabetes induces cognitive dysfunction in rats associated with alterations of the gut microbiome and metabolomes in serum and hippocampus	Metabolomics	NMR-based metabolomic analysis	STZ-induceddidiabetic rats	Explored the role of the gut microbiota in DE.
Song L. et al. (56) Urine Metabonomics Reveals Early Biomarkers in Diabetic Cognitive Dysfunction	Metabolomics	UPLC-Q/TOF-MS	STZ-induced diabetic mice	Metabolites such as niacinamide, pyroglutamic acid, and sphinganine were found to be significantly altered in mice with early cognitive dysfunction.
Qi et al. (57) Liraglutide reduces oxidative stress and improves energy metabolism in methylglyoxal-induced SH-SY5Y cells	Metabolomics	NMR	SH-SY5Y cells	Liraglutide reduces oxidative stress and improves energy metabolism
Zhou et al. (20) Comprehensive transcriptomic analysis indicates brain regional specific alterations in type 2 diabetes	Transcriptomics	RNA-Seq	Postmortem human brain tissue	Region-specific transcriptomic alterations
Bury et al. (58) Type 2 diabetes mellitus-associated transcriptome alterations in cortical neurones and associated neurovascular unit cells in the ageing brain	Transcriptomics	mRNA microarray	Post mortem human cortical neurons, astrocytes and endothelial cells	Transcriptomic changes in cortical neurons
Rahman et al. (59) Network-Based Bioinformatics Approach to Identify Molecular Biomarkers for Type 2 Diabetes that Are Linked to the Progression of Neurological Diseases	Transcriptomics	Gene expression analysis	Gene expression datasets from control and T2D individuals	197 DEGs common to T2D and neurological diseases
Song X, et al. (60) Multi-Omics Characterization of Type 2 Diabetes Mellitus-Induced Cognitive Impairment in the db/db Mouse Model.	Multi-omics	RNA-Seq 16S rDNA seq UPLC-Q/TOF-MS	db/db mice	Complex interactions within the brain-gut axis mediated disturbances in mitochondrial metabolism, bile acid and steroid metabolism, and GPCR signaling

db/db, homozygous mutation of the leptin receptor; HFD, high-fat diet; SD, Sprague- Dawley; STZ, streptozotocin; GPCR, G Protein-Coupled Receptor; ↑, upregulation; ↓, downregulation.

## 2 Omics analyses of DE in animal models

### 2.1 Transcriptomics

Omics research has opened powerful new avenues for understanding the complexities of diabetic encephalopathy (DE) in animal models. By examining the intricate changes in gene expression, these studies are painting a clearer picture of how this debilitating condition develops and progresses (20).

Several studies have utilized RNA sequencing (RNA-Seq) to identify differentially expressed genes (DEGs) in various brain regions of diabetic animal models. For instance, Han et al. performed RNA-Seq to characterize the hippocampal transcriptome in diabetic dogs, providing valuable insights into the disease in a clinically relevant model. The hippocampus, a brain region crucial for learning and memory, was examined. Gene expression changes related to metabolic disorders and cognitive dysfunction were explored, with altered expression of genes such as *DBH* (dopamine beta-hydroxylase, an enzyme essential for noradrenaline synthesis, a neurotransmitter involved in attention and arousal), *IGFBP2* (insulin-like growth factor-binding protein 2, a protein involved in neuronal survival and growth), *AVPR1A*(arginine vasopressin receptor 1A, a receptor involved in social memory, stress response and blood pressure), and *DRAXIN* (dorsal repulsive axon guidance protein, a protein involved in axonal guidance and neurogenesis, the process of generating new neurons), all of which are involved in hippocampal function and memory processes. These changes suggest potential mechanisms for cognitive decline in type 1 diabetes mellitus (T1DM) (27).

Liu et al. also highlighted the utility of high-throughput RNA sequencing (RNA-Seq) in identifying the circRNA (circular RNA) expression profile in DE. In primary hippocampal neurons of db/db mice induced with advanced glycation end products (AGEs), the expression of three circRNAs showed significant changes. Among these, circ-Nbea (circular RNA derived from the neurobeachin gene), potentially functioning as a miRNA sponge (a molecule that regulates gene expression by binding to microRNAs), was the most dramatically reduced, suggesting a potential key role in the pathogenesis and progression of DE (28).

Qin et al. applied RNA-Seq to analyze gene expression in neural stem cells of a high-fat diet (HFD) and streptozotocin (STZ)-induced type 1 diabetes mellitus (T1DM) mouse model treated with the Taohe Chengqi decoction. This transcriptomic approach allowed for the identification of changes in gene expression profiles related to the improvement of cellular senescence, a process that plays a crucial role in the cognitive decline associated with diabetes (29). Furthermore, studies have demonstrated that HFD affects miRNA expression in the hippocampus, leading to changes in the levels of proteins involved in neuroplasticity and synaptic function, including *SYT1* (synaptotagmin 1, involved in neurotransmitter release), *CaMK1D* (calcium/calmodulin-dependent protein kinase ID, involved in synaptic plasticity), *GRIN2B* (glutamate ionotropic receptor NMDA type subunit 2B, a key component of NMDA receptors, crucial for learning and memory), *SATB2* (special AT-rich sequence-binding protein 2, involved in chromatin remodeling and gene regulation in neurons), and *NOVA1* (neuro-oncological ventral antigen 1, an RNA-binding protein involved in alternative splicing and neuronal function) (61).

In addition to hyperglycemia, hypoglycemia also contributes to cognitive dysfunction. Specifically, Castillo-Armengol et al. employed single-nuclei RNA-seq to analyze changes in the hypothalamic transcriptome of diabetic mice exposed to recurrent hypoglycemia (30). This approach allowed for a global view of gene expression changes at the hypothalamic level in response to hypoglycemic episodes. Gene ontology analysis highlighted a decrease in genes involved in oxidative phosphorylation and the expression of vacuolar H+- and Na+/K+-ATPases.

Transcriptomic approaches were not only applied to neurons but also to other important cerebral cells like microglial cells. Ma et al. revealed significant microglial activation and a pro-inflammatory phenotype in db/db mice using scRNA-seq analysis, emphasizing the critical role of neuroinflammation in the pathogenesis of diabetes-associated cognitive dysfunction (DCD) (31).

Neuroinflammation emerges as a key player in DE, with increased expression of inflammatory cytokines. Wang et al. investigated the miR-384-5p/Beclin-1 regulatory axis (a signaling pathway that involves autophagy and stress response), previously identified in atherosclerosis, to explore its role in macrophage autophagy, which seems to have a harmful effect in diabetic encephalopathy (32).

Oligodendrocyte precursor cells (OPCs) are another important cellular component in the neuroplasticity regulation in DE. Xiang et al. performed single-cell RNA sequencing in the hippocampus of a diabetic-induced cognitive impairment (CI) animal model. They identified five distinct sub-clusters of OPCs with unique gene expression profiles (33). Notably, they observed an overexpression of somatostatin receptor 2 (SSTR2, a receptor that modulates neurotransmitter release and neuronal excitability) in a sub-cluster of OPCs of CI mice. SSTR2 activation is associated with neuroactive ligand-receptor interactions, axon regeneration, and mTOR/FoxO/PI3K-Akt/cAMP signaling pathways (pathways fundamental for the survival and growth of neurons, involved in cell growth, survival, and metabolism). Furthermore, DEGs of the OPCs sub-cluster indicated an upregulation of genes involved in neuronal development and inflammatory processes in animals with CI. The study aims to explore how synaptic dysfunction and synaptic plasticity are affected by this pathological condition and how these changes may be linked to alterations in gene expression (34).

Tau hyperphosphorylation is another crucial pathological feature in DE. Qu et al., using a Rat m6A-mRNA epitranscriptomic microarray, demonstrated that high glucose inhibits ALKBH5-mediated Dgkh m6A demethylation, leading to tau hyperphosphorylation through PKC-α activation (21). This study observed elevated levels of p-tau (T231) and p-tau (PHF-1) in the hippocampus of STZ-induced diabetic rats, consistent with cognitive decline (21). Moreover, Shi et al. demonstrated that decreased miR-132 plays a crucial role in DE by regulating the GSK-3β/Tau pathway (a signaling pathway involved in tau phosphorylation), suggesting its potential as a therapeutic target. Furthermore, miR-132 has been reported to be reduced in type 2 diabetes mellitus (T2DM) islets and gestational diabetes, suggesting its potential role as a biomarker (34).

An interesting result obtained with transcriptomic analyses is the increased expression of lncRNA V-16 in the hippocampus of diabetic rats. This lncRNA acts as a sponge on miR-205, which in turn increases Gnb3 (guanine nucleotide-binding protein subunit beta 3). Gnb3 is a subunit of G-proteins involved in intracellular signal transduction, and its overexpression in mice can induce obesity and metabolic syndrome (35). This chain reaction might be one of the ways diabetes causes cognitive problems. This discovery could lead to new ways to treat or prevent these problems.

To explore the pathogenesis of DE, Fu et al. studied the competitive endogenous RNA (ceRNA) network involving lncRNA-miRNA-mRNA interactions in a diabetic encephalopathy model based on GK-/- diabetic rats. This study proposes that ceRNA interactions linking lncRNAs NONRATT007456.2, NONRATT020546.2, miR-5132-5p, and MRC1 (mannose receptor C-type 1) contribute to the inflammation-driven pathogenesis of diabetic encephalopathy (36).

### 2.2 Proteomics

Several studies in the last decade have used proteomic approaches to research neurological complications of diabetes mellitus, focusing on diabetic encephalopathy (DE), a condition characterized by mild to moderate cognitive decline.

One of the main applications of proteomics in the study of diabetic encephalopathy is the search for potential biomarkers for diabetes-related brain changes.

In a 2011 study, Li X. et al. compared global brain protein profiles between rats with type 1 diabetes and healthy control rats using a 2-DE followed by MALDI-TOF-MS approach. This analysis identified 8 differentially expressed proteins, with myosin light chain kinase (MLCK) showing the greatest change. The authors suggest that over-expression of MLCK may lead to excessive contraction of vascular smooth muscle, exacerbating ischemia and potentially contributing to vascular dementia (37).

Also other proteins such as calbindin, creatine kinase B-type (B-CK), heat shock protein 60 (HSP60), heat shock protein 71 (HSP71), ATP synthase, cyclin-G and pantothenate kinase (PANK1) were differentially expressed between T1DM and normal rats. These proteins are implicated in a variety of crucial cellular processes, including neuronal function and cell cycle regulation, energy metabolism, and the cellular response to stress and injury (37).

Recognizing the impact of diabetes on neuronal energy metabolism, Taurino F. et al. conducted a proteomic analysis with a specific focus on mitochondrial Complex I of the respiratory chain in streptozotocin-induced T1DM rats (38). Using 2D-DIGE to separate and visualize proteins, followed by LC-MS/MS for identification, they discovered a significant reduction in the NADH:ubiquinone oxidoreductase core subunit S3 (Ndufs3). This finding underscores the role of mitochondrial dysfunction in the development of diabetes-related neurological complications and points towards potential therapeutic targets for managing this condition (38).

Another crucial energy source for neurons is lactate. Zhang N. et al. investigated the impact of human islet amyloid polypeptide (hIAPP), a peptide implicated in the pathogenesis of type 2 diabetes, on lactate transport in the brain (39).

Lactate transport is mediated by monocarboxylate transporters (MCTs), which require accessory proteins like CD147 and gp70 for proper function.

Researchers analyzed the effects of continuous hIAPP stimulation on the membrane proteins of murine cell culture of oligodendrocytes (OLs) and neurons themselves. They identified protein Yip1 Domain Family Member 2 (YIPF2) that, in the presence of hIAPP, binds to CD147 in OLs, competing with MCT1. So hIAPP disrupts lactate transport in the brain by reducing the interaction between MCT1 and CD147 in OLs. These findings suggest a novel mechanism by which hIAPP may contribute to neuronal dysfunction in type 2 diabetes (39).

In another proteomic study, this time employing affinity chromatography and peptide mass fingerprinting via MALDI-TOF-MS, Calábria L.K. et al. isolated and investigated calmodulin-binding proteins (CaMBPs) in the hippocampus of rats with streptozotocin-induced type 1 diabetes and healthy controls. CaMBPs are involved in a variety of cellular functions, including calcium signaling, cytoskeletal organization, and gene transcription. The researchers firstly found that several CaMBPs were differentially expressed in the diabetic brain compared to the healthy brain, like miosin II-B. Some proteins were exclusively present in the diabetic brain, while others were only found in the healthy brain, suggesting that diabetes alters the profile of these proteins (40).

The molecular causes of diabetic encephalopathy (DE) are still not fully defined, but they are certainly associated with a pro-inflammatory condition.

In diabetes, chronic hyperglycemia can lead to the formation of endogenous Advanced Glycation End Products (AGEs) through various pathways, such as the polyol pathway or the peroxidation of polyunsaturated fats. AGEs can increase cellular oxidative stress by binding to their cellular receptors (RAGE), by stimulating the activation of nuclear factor kappa-light-chain-enhancer of activated B cells (NF-kB) and JNK (c-Jun N-terminal Kinase), which promote the production of reactive oxygen species (ROS) (62).

In 2010, LU M. et al., highlights the importance of AGEs/RAGE/NF-kB p65 pathway in the development of diabetic encephalopathy and suggests that grape seed proanthocyanidin extract (GSPE), a natural antioxidant, could protect against brain injury in diabetic rats (41).

In some studies, the efficacy of some molecules in the treatment of DE has been evaluated. Yu W. et al. investigated the neuroprotective effects of dl-PHPB (potassium 2-(1-hydroxypentyl)-benzoate) in KK-Ay mice, a murine model of hyperglycemia. The dl-PHPB is a novel drug candidate for ischemic stroke that has been shown to prevent neuropathological changes, inhibit oxidative damage, and reduce inflammatory reactions (42). The drug modulates the PI3K/Akt/GSK-3β signaling pathway, critical for glucose metabolism and tau protein phosphorylation, both of which are disrupted in diabetes and Alzheimer’s disease. Using 2D-DIGE (two-dimensional difference gel electrophoresis) and LC-LTQ-MS/MS analysis, the authors compared protein expression in the brains of mice treated with dl-PHPB to those that were not treated. This analysis revealed several altered proteins in the cortex and hippocampus, with 14 identified in cortical tissue and 11 in hippocampal tissue. Notably, dl-PHPB improved memory in this T2DM mouse model. Importantly, the effects of dl-PHPB were independent of blood glucose levels, suggesting it could be a potential treatment for diabetes-associated cognitive deficits (42).

Trichostatin A (TSA) is another molecule that has demonstrated protective effects on the central nervous system in Alzheimer’s disease (AD) and hypoxic-ischemic brain injury. In a recent study, Wei W. et al. treated spontaneous type II diabetic mice (db/db) and control mice (db/m) with TSA (43). A proteomics approach was employed to analyze hippocampal proteins, which revealed 174 differentially expressed proteins between the two groups. TSA treatment restored the expression of 111 of these proteins, many of which are involved in critical processes such as longevity regulation, insulin signaling, peroxisome function, endoplasmic reticulum protein processing, and ribosomal biogenesis (43). Notably, catalase (CAT) and protein kinase AMP-activated catalytic subunit alpha 2 (PRKAA2), enzymes crucial for oxidative stress response and cellular energy regulation, were significantly reduced in the hippocampus of the diabetic mice and TSA treatment effectively restored the expression of these enzymes (43).

Proteomic approach was used not only to investigate the effect of possibly therapeutic molecules on DE animal models, but also to understand if substances used in diagnostic workup had an toxic effect on the brain. Therefore, Gd-based contrast agent (GBCA): gadodiamide, gadopentetate dimeglumine and gadoterate meglumine, are contrast media used to enhance tissue visualization for magnetic resonance and Wang S. et al. studied Gadolinium (Gd) retention and elimination in the brain of diabetic rats after multiple administrations of GBCA (44). Inductively coupled plasma mass spectrometry (ICP-MS) analysis quantified Gd in brain tissues, and surprisingly, was found that less GBCA accumulated in the brains of diabetic rats compared to healthy rats. It seems that diabetic rats clear the contrast agent from their system more quickly and these findings suggest that diabetes might actually reduce the retention of this contrast agent in the brain, possibly due to changes in the blood-brain barrier and faster elimination from the body (44).

Another interesting study, Diviccaro et al., used a proteomic approach to investigate the correlation between neuroactive steroids, gut microbiota and stress hormones in the development of diabetic encephalopathy in the Zucker diabetic fatty (ZDF) rat model of T2D (45). Also was found a neuroactive steroid imbalance and gut microbiota dysbiosis. These results highlights a particular reduction of allopregnanolone (ALLO) in the hippocampus linked to specific gut bacteria that may influence both neuroactive steroid levels and cognitive function (45). This research suggests potential therapeutic avenues, such as targeting ALLO, dehydroepiandrosterone, or specific gut bacteria to protect against cognitive decline in type 2 diabetes (45).

The use of proteomic approaches has allowed the study of how diabetes impacts the proteome of brain cells in DE animal models. It has been highlighted that the diabetic condition was associated with memory impairment, increased oxidative stress, neuroinflammation, increased corticosterone levels, and signs of mitochondrial dysfunction.

### 2.3 Lipidomics

To gain a more holistic understanding of how cells function and how diseases disrupt these processes, researchers combined proteomic and lipidomic approaches. Lipidomic is a rapidly growing field that studies the complete set of lipids (the lipidome) within a biological system. It holds immense potential for understanding biological processes and identifying new biomarkers and drug targets.

The techniques used to identify and quantify a wide range of lipid species are HPLC and GC couple to mass spectrometry, including tandem MS (MS/MS), time-of-flight MS (TOF-MS), and ion trap MS (63).

The lipidome is incredibly diverse, with thousands of lipid species varying in structure and function. Lack of standardization in lipidomic techniques can make it difficult to compare results across different studies.

The most important applications of lipidomic are biomarker discovery, nutrition research, basic research and diagnosis of metabolic diseases (64).

Also in the study of diabetic complications, like enecephalopaty, a lipidomic approach was used to analyze myelin in the cerebral cortex of diabetic rats induced by streptozotocine. Cermenati G. et al, in 2017, quantified total fatty acid in rats cerebral cortex’s myelin by flow injection analysis-tandem mass spectrometry (FIA-MS/MS) method and free cholesterol was quantified by GC-MS (46). They found that diabetes significantly alters the lipid composition of myelin, particularly decreasing levels of phospholipids, plasmalogens and cholesterol. These changes may contribute to myelin dysfunction and damage. However, treatment with dihydroprogesterone (DHP), a neuroactive steroid, was able to restore myelin lipid and protein profiles to those of non-diabetic rats. This suggests that DHP may have therapeutic potential for protecting myelin and preventing or treating diabetic encephalopathy (46).

### 2.4 Metabolomics

To overcome the limitations of current knowledge regarding the pathogenetic mechanisms of DE, scientific research has increasingly focused on omics approaches, with particular emphasis on metabolomics. The following sections present several studies employing metabolomics in the investigation of DE, providing novel insights into its pathogenesis and paving the way for the identification of potential biomarkers and therapeutic targets.

Studies conducted on animal models of DE have yielded crucial insights into pathogenic mechanisms and potential therapeutic interventions. A recurring theme is the disruption of cerebral energy metabolism coupled with elevated oxidative stress. Several studies have highlighted alterations in key metabolites involved in these processes. For instance, mass spectrometry imaging analyses, as reported in several studies (47–49), have identified significant changes in multiple metabolites. In particular, glucose-6-phosphate, glutamine, adenosine, and L-carnitine varied in different brain regions, including the hippocampus, cortex, and thalamus. These alterations suggest dysfunctions in glucose metabolism, with glucose-6-phosphate accumulation potentially resulting in ATP reduction and pentose phosphate pathway activation. The alteration of the glutamate-glutamine cycle causes glutamine accumulation and acido γ-aminobutirrico (GABA) reduction, with consequent neuroinflammation and impaired neurotransmission. Lipid metabolism and aspartate levels were also altered. The outcome of all this is impaired energy production and an increase in oxidative stress. Notably, as demonstrated by Cheng et al., salidroside treatment can positively modulate these metabolic alterations, supporting its therapeutic potential (48).

Another important aspect emerging from the studies is the role of oxidative stress and oxidative damage at a regional level. As reported by Meng et al., focusing on the mapping of fatty aldehydes (FALs) in the brains of diabetic rats using mass spectrometry imaging techniques, a significant increase in these molecules, markers of oxidative stress, was found in vulnerable brain areas such as the hippocampus, correlating with cognitive deficits (49). This finding is consistent with the observations of Chen et al., who identified a specific metabolic signature in the hippocampus of streptozotocin-induced DE rats, characterized by a reduction in N-acetyl aspartate and dihydroxyacetone phosphate, and an increase in homocysteine and glutamate (50). These hippocampal metabolic alterations suggest the presence of neurotoxicity and energy dysfunction in this brain region crucial for memory and learning.

Other studies investigated the impact of diabetes on the neuroendocrine system and neurosteroidogenesis (51, 52). As highlighted by Romano et al., a significant reduction in neurosteroids such as pregnenolone and allopregnanolone was observed in the hippocampus and prefrontal cortex of diabetic rats, suggesting an alteration of neuroendocrine pathways that could contribute to early cognitive dysfunction (51). Pesaresi et al. further explored this aspect, finding significant differences between males and females in the levels of neuroactive steroids, including dehydroepiandrosterone (DHEA) and allopregnanolone, highlighting a differential vulnerability to DE between the sexes (52).

Finally, several studies have evaluated the efficacy of therapeutic interventions. Some studies have investigated the effects of Huang-Lian-Jie-Du Decoction (HLJDD), a traditional Chinese medicine formulation on DE animal models (53, 54). It has been discovered that HLJDD improves cognitive function, attenuates inflammation, and reduces oxidative stress, with a regulation of phospholipid, fatty acid, and glucose metabolism. Specifically, as shown by Zhuang et al., HLJDD acts through the inhibition of the AGEs/RAGE/NF-κB pathway and normalizes 12 of the 18 altered metabolites (53). Furthermore, an untargeted metabolomic analysis performed by He et al. shows that HLJDD restores the levels of 11 key metabolites involved in processing fats, glucose, and antioxidants (54).

An innovative approach was adopted by Gao et al., who explored the role of the gut microbiota in DE (55). This study found significant alterations in the gut microbiota of diabetic rats, associated with a reduction in the levels of TCA cycle metabolites and amino acid metabolism in the hippocampus, suggesting an influence of the microbiota on brain metabolism and cognitive decline (54). Song et al., conversely, identified urinary metabolites (niacinamide, pyroglutamic acid, and sphinganine) as potential early biomarkers of diabetic cognitive dysfunction, highlighting alterations in nicotinamide and tryptophan metabolism pathways (56).

## 3 Omics analyses of DE *in vitro* models

Currently, omics approaches have been infrequently applied to cellular models of DE. The *in vitro* study reported in this review, using SH-SY5Y neuronal cell models, allowed for the investigation of the mechanisms of action of specific therapeutic interventions at the cellular level, particularly the effect of liraglutide. As reported by Qi et al., the study focused on the ability of liraglutide to counteract oxidative stress and improve energy metabolism in a context of methylglyoxal 1qz(MG)-induced damage (57). MG, a toxic byproduct of glucose metabolism, is known to induce oxidative damage and impair neuronal functions. As demonstrated by a metabolomics analysis based on 1H nuclear magnetic resonance, liraglutide significantly reduces ROS (reactive oxygen species) levels in MG-treated SH-SY5Y cells. This effect is associated with an improvement in oxidative phosphorylation and gluconeogenesis, metabolic processes crucial for cellular energy production. In particular, an increase in the activity of superoxide dismutase (SOD), a key antioxidant enzyme, was observed, with this increase being directly correlated with the reduction in oxidative damage (57). Furthermore, liraglutide treatment led to improved ATP production, suggesting enhanced mitochondrial efficiency and a healthier cellular energy balance. The observed metabolic changes were thus associated with an improved cellular energy balance and an overall reduction in oxidative damage (57).

In summary, the *in vitro* study demonstrated that liraglutide exerts a protective effect on SH-SY5Y neurons exposed to MG, counteracting oxidative stress and improving energy metabolism through increased antioxidant activity and optimized ATP production. This targeted action on neuronal energy metabolism supports the therapeutic potential of liraglutide in the context of DE.

## 4 Omics analyses of DE in human cells

Studies on animal models provide an overview of the metabolic alterations in the brain caused by diabetes. But the limitation is work on an organism different from humans, with a different genome. This is why it is necessary to study DE also in human cells, even if they are very difficult to obtain. Zhou et al. (2019) have worked on postmortem human brain tissue, focusing on areas such as the hippocampus, cortex, and striatum. Region-specific transcriptomic alterations were identified, with the hippocampus showing significant dysregulation of genes involved in synaptic function and memory formation, while the cortex exhibited changes related to vascular regulation and inflammation (20).

Also Bury et al. (2021) worked on human cells, in particular on neurons, astrocytes and endothelial cells obtained by immuno-laser capture microdissection of the temporal cortex of 6 T2D patients with impaired cognitive function and 6 control (58). A mRNA microarray analysis investigated transcriptomic changes in cortical neurons and associated neurovascular unit cells in the aging brain of individuals with T2D. The study revealed alterations in insulin signaling pathways, the cell cycle, cellular senescence, inflammatory mediators, and mitochondrial respiratory electron transport chain components (58).

Furthermore, to bypass the limited availability of human samples, Rahman et al. (2020) used gene expression transcriptomic datasets from control and disease-affected individuals to identify differentially expressed genes (DEGs) in tissues of patients with T2D and neurological diseases (NDs) (59). They identified 197 DEGs common to both T2D and ND datasets. The study highlights the potential of network-based approaches in identifying shared molecular pathways between T2D and NDs, providing insights into the pathogenic links between these conditions (59).

## 5 Discussion

**Figure 2:**
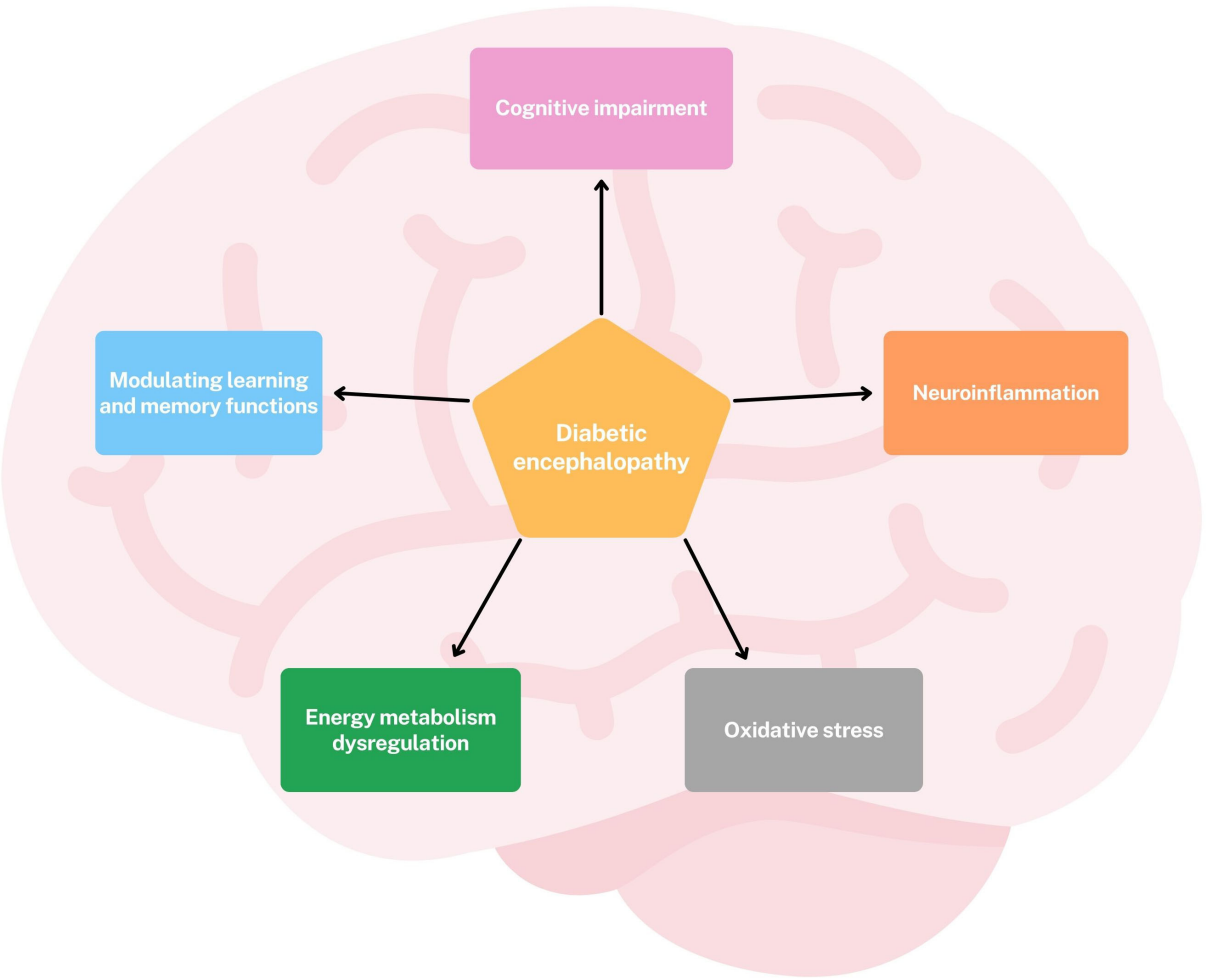
Main alterations found in DE through omics approaches.

**Figure 3:**
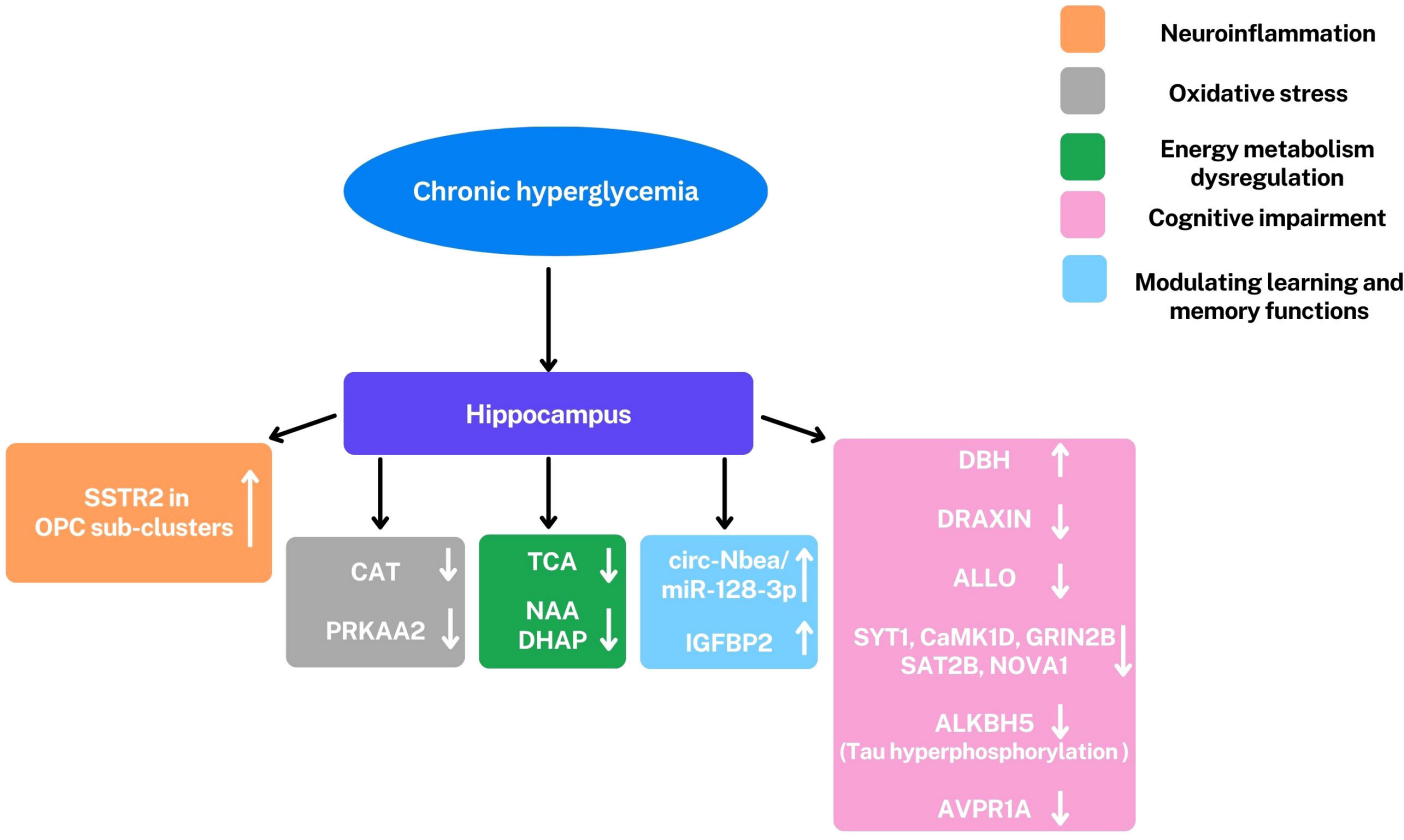
Representation of principal alterations observed in the hippocampus under hyperglycemic conditions through omics analyses. The most significant observed alterations involve a series of factors: Somatostatin Receptor 2 (SSTR2), circ-Nbea/miR-128-3p, insulin-like growth factor binding protein-2 (IGFBP2), dopamine β-hydroxylase (DBH), DRAXIN, Allopregnanolone (ALLO), Synaptotagmin 1 (SYT1), calcium/calmodulin dependent protein kinase I delta (CaMK1D), 2B subunit of N-methyl-D-aspartate glutamate receptor (GRIN2B), 2B subunit of N-methyl-D-aspartate glutamate receptor (GRIN2B) and Neuro-oncological ventral antigen 1 (NOVA1), ALKBH5, arginine vasopressin receptor 1A (AVPR1A), CAT, PRKAA2, tricarboxylic acid cycle (TCA), NAA and dihydroxyacetone phosphate (DHAP); ↑,upregulation; ↓, downregulation. These alterations are involved in cognitive impairment (pink), modulation of learning and memory (light blue), neuroinflammation (orange), oxidative stress (gray), and dysregulation of energy metabolism (green). All these conditions are characteristics of DE. Oligodendrocyte precursor cells (OPCs).

Omics approaches have provided valuable new insights into the pathogenesis and potential therapeutic targets of diabetic encephalopathy (DE). Leveraging these technologies has moved our understanding beyond a traditional focus on oxidative stress, revealing that DE is a multifactorial condition characterized by oxidative stress, dysregulation of cerebral energy metabolism, neuroinflammation, cognitive impairment, and modulation of learning and memory functions. (**Figure 2**) Transcriptomics studies have highlighted significant dysregulation of gene and protein expression in the hippocampus, a brain region crucial for learning and memory. Significant alterations have been observed in genes involved in neuroplasticity and synaptic function, including: *DBH*, an enzyme essential for noradrenaline synthesis, a neurotransmitter involved in attention and arousal; *IGFBP2*, a protein involved in neuronal survival and growth; *AVPR1A*, a receptor involved in social memory, stress response and blood pressure; *DRAXIN*, a protein involved in axonal guidance and neurogenesis, the process of generating new neurons (27) (**Figure 3**).

These genetic alterations, combined with evidence of circRNA dysregulation, disrupt the complex processes that underlie synaptic plasticity, ultimately contributing to the synaptic dysfunction and cognitive decline characteristic of DE.

**Figure 4:**
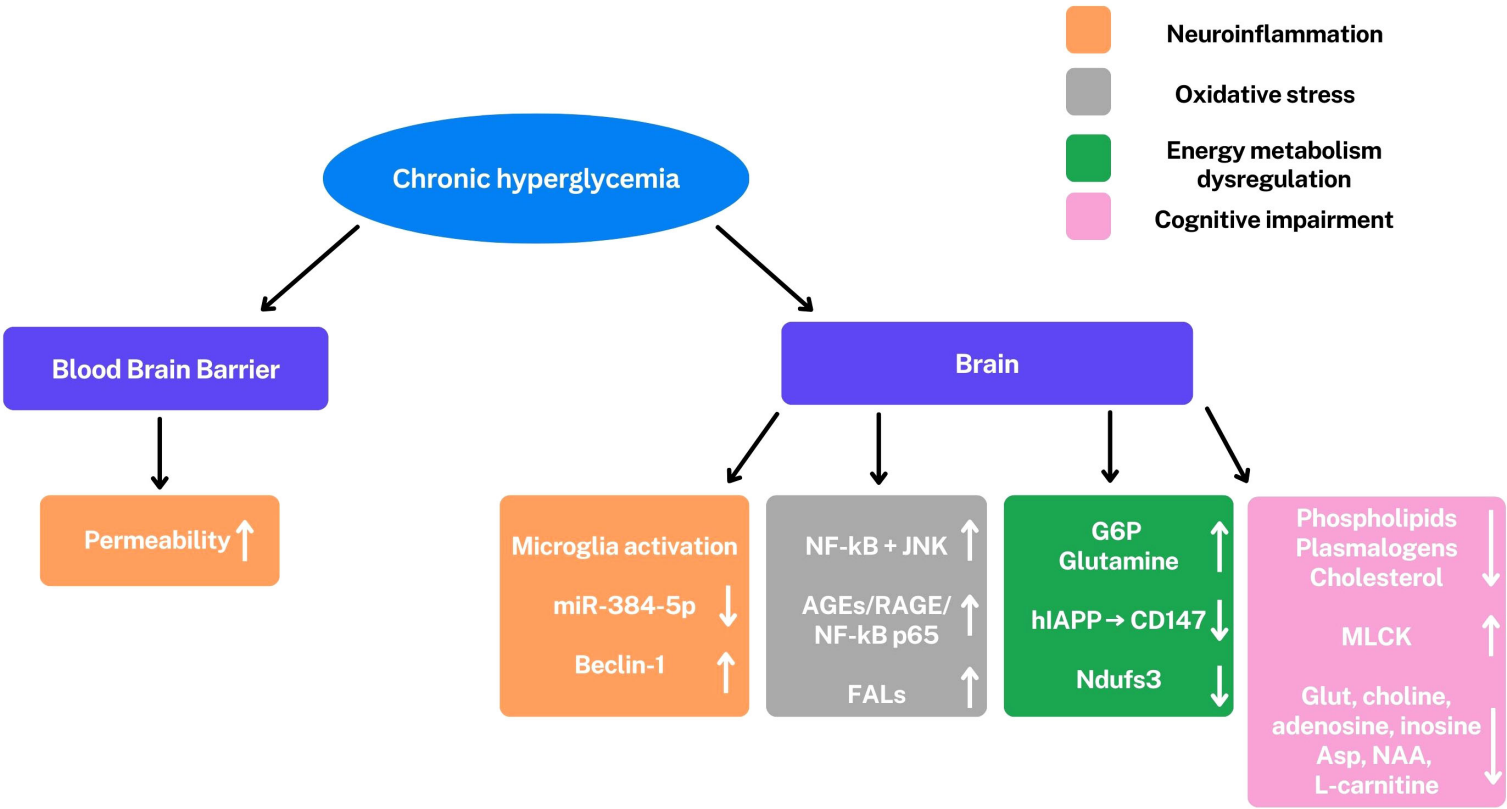
Under hyperglycemic conditions, there is a permeability increase of the blood-brain barrier and an activation of microglia. A series of altered pathways and molecules are observed: miR-384-5p/Beclin-1, nuclear factor kappa-light-chain-enhancer of activated B cells (NF-kB), c-Jun N-terminal Kinase (JNK), PI3K/Akt/GSK-3β signaling, Fatty aldehydes (FALs), glucose-6-phosphate (G6P), glutamine, human islet amyloid polypeptide (hIAPP) reduced CD147, Ndufs3, phospholipids, plasmalogens, cholesterol, glutamate (Glut), adenosine, inosine, aspartate (Asp), N-acetyl aspartate (NAA), L-carnitine and myosin light chain kinase (MLCK). ↑,upregulation; ↓, downregulation. These alterations are involved in cognitive impairment (pink), modulation of learning and memory (light blue), neuroinflammation (orange), oxidative stress (gray), and dysregulation of energy metabolism (green). All these conditions are characteristics of DE.

Microglial activation and increased pro-inflammatory cytokines, underscore the central role of neuroinflammation in DE (**Figure 4**).

These conditions are mediated by the miR-384-5p/Beclin-1 axis, a signaling pathway that involves autophagy and stress response (32). This inflammatory response contributes to neuronal dysfunction and cognitive impairment.

In addition to neuroinflammation, alterations in energy metabolism also play a critical role in neuronal damage. Metabolomic studies have revealed a profound dysregulation of cerebral energy metabolism, with alterations in the levels of key metabolites such as glucose-6-phosphate, glutamine and adenosine. The accumulation of glucose-6-phosphate suggests an alteration in glycolysis, while variations in the glutamate-glutamine cycle indicate an interruption in neurotransmission, the communication between neurons. The increase in fatty aldehydes, indicators of lipid oxidative stress, in vulnerable regions like the hippocampus, further highlights the presence of neuronal damage. Neuronal energy deficit is further exacerbated by the disruption of lactate transport, an important energy source for neurons, caused by hIAPP (human islet amyloid polypeptide), a peptide involved in the pathogenesis of T2DM. At the protein level, the reduction of the Ndufs3 subunit of mitochondrial complex I, a crucial component of the electron transport chain involved in ATP production, confirms mitochondrial dysfunction and impaired ATP synthesis.

Furthermore, transcriptomic analyses have shown that the increase in lncRNA V-16 and altered expression of circRNA species, such as circ-Nbea, highlight the crucial role of non-coding RNA species in DE. These molecules, which do not code for proteins, can modulate gene expression and contribute to the pathogenesis of the disease through complex mechanisms, such as the regulation of miRNAs.

Tau protein hyperphosphorylation, mediated by PKC-α, results in the formation of neurofibrillary tangles, a pathological hallmark of DE and Alzheimer’s disease. This process is intricately linked to the alteration of the ALKBH5 enzyme, an RNA demethylase that modulates mRNA demethylation.

Additionally, lipidomic studies have revealed a reduction in phospholipids, plasmalogens, and cholesterol in myelin, compromising the structural and functional integrity of myelin sheaths, thereby impairing neurotransmission.

Cognitive dysfunction may also be associated with reduced neurosteroid levels, such as pregnenolone and allopregnanolone, highlighting a dysregulation of neuroendocrine pathways.

The use of common diabetes drugs, such as liraglutide, has demonstrated a reduction of ROS production and oxidative damage *in vitro* in human neuronal cells under hyperglycemic conditions. This may be linked to the pleiotropic effects of glucagon-like peptide-1 (GLP-1) receptor agonists, which include increased tissue sensitivity to insulin and reduced glucagon production (57).

Additionally, some studies on the gut microbiome introduce an innovative perspective, suggesting that interactions between the microbiota and brain metabolism play a crucial role in the pathogenesis of DE. Using a multi-omics approach, Song X. et al. (2022) investigated the mechanisms linking T2DM to cognitive deficits. A complex network of interactions between the brain, gut, and systemic metabolism was discovered. In particular, bile acid and steroid metabolism emerges as a crucial factor. The observed alterations, such as increased taurine and cholesterol levels, indicate a key role for the interaction between the microbiota, metabolites, and the brain. Bile acids, modulated by the gut microbiota, act as signaling molecules that influence brain function (60). Alterations in the gut microbiome correlate with variations in neurotransmitter metabolites and those of the TCA (tricarboxylic acid) cycle. This suggests a potential role for gut microbiota modulation, such as with probiotics and prebiotics to induce the growth of beneficial bacteria, which could represent a new target for therapeutic interventions in DE.

Despite considerable advancements in elucidating the molecular mechanisms of DE, several challenges persist. The heterogeneity of DE, characterized by diverse clinical manifestations and underlying pathophysiological alterations, presents a significant obstacle. This variability, significantly amplified by comorbidities associated with metabolic syndrome beyond diabetes mellitus, such as cardiovascular disease, non-alcoholic fatty liver disease (NAFLD), and dyslipidemia, complicates the identification of universally applicable biomarkers and therapeutic targets (65).

Another challenge lies in the limited availability of human studies. While animal models offer valuable insights, translating these findings to human populations necessitates further validation. Increased efforts are required to investigate the molecular mechanisms of DE in human subjects, particularly during the early stages of the disease.

Furthermore, the integration of multi-omics approaches, encompassing transcriptomics, metabolomics, and proteomics, holds immense potential for a more comprehensive understanding of DE. However, realizing this potential necessitates the development of standardized protocols and robust data analysis methods, and future studies could integrate multiple methodologies for a more comprehensive analysis.

Finally, translating research findings into effective clinical practice remains a formidable challenge. While promising therapeutic targets have been identified, further research is necessary to develop safe and efficacious treatments for DE.
